# Effect of Supplementation of a Whey Peptide Rich in Tryptophan-Tyrosine-Related Peptides on Cognitive Performance in Healthy Adults: A Randomized, Double-Blind, Placebo-Controlled Study

**DOI:** 10.3390/nu10070899

**Published:** 2018-07-13

**Authors:** Masahiro Kita, Kuniaki Obara, Sumio Kondo, Satoshi Umeda, Yasuhisa Ano

**Affiliations:** 1 Research Laboratories for Health Science & Food Technologies, Kirin Company, Ltd., Yokohama 1-13-5, Japan; k-obara@kirin.co.jp (K.O.); Yasuhisa_Ano@kirin.co.jp (Y.A.); 2 Kensyokai Medical Corporation, Osaka 2-12-16, Japan; s.kondo@drc-web.co.jp; 3 Department of Psychology, Keio University, Tokyo 2-15-45, Japan; umeda@flet.keio.ac.jp

**Keywords:** dairy food, whey peptide, cognitive function

## Abstract

**Background:** Previous epidemiological and clinical studies have shown that dairy products have beneficial effects on cognitive decline and dementia. Enzymatic digestion of whey protein produces a whey peptide rich in tryptophan-tyrosine-related peptides which improve cognitive performance in mice. We evaluated the effects of whey peptides on cognitive functions in healthy adults in a randomized, double-blind, placebo-controlled design. **Methods:** 101 healthy adults (45 to 64 years), with a self-awareness of cognitive decline received either whey peptide or placebo supplements for 12 weeks. Changes in cognitive function were assessed using neuropsychological tests at 6 and 12 weeks after the start of supplementation. **Results:** Verbal fluency test (VFT) score changes tended to be higher in the whey peptide group compared with the placebo at 12 weeks. Subgroup analysis classified by the degree of subjective fatigue showed that changes in the VFT as well as the Stroop and subjective memory function tests between baseline and 6 weeks of intervention were significantly better in subjects with high-level fatigue from the whey peptide group as compared to the placebo group. Conclusions: Intake of whey peptide might improve cognitive function in healthy middle- and older-aged adults with high subjective fatigue levels. Further studies will elucidate the relationship among cognitive improvement, whey peptides, and psychological fatigue.

## 1. Introduction

With the rapid increase in the world’s aging population, the number of people suffering with dementia and cognitive decline is rapidly increasing. The United Nations estimates that the number of people aged over 60 years will reach 1.4 billion by 2030, and 2.1 billion by 2050 [1]. The number of patients with dementia is estimated to reach 130 million by 2050 [2]. At present, there is no effective therapy for dementia, and thus preventive approaches are receiving increasing attention.

Recent epidemiological and clinical studies have suggested that consumption of dairy products, including yogurt and cheese, may reduce the risk of cognitive decline in later life [3]. A prospective cohort study surveyed more than 1000 Japanese subjects in a local community and showed that frequent intake of milk and dairy products is associated with a lower risk of cognitive decline and dementia [4]. In addition, a retrospective cross-sectional study in Australia revealed that intake of low-fat dairy products is beneficial for social functioning and memory function [5]. A clinical trial using a sample of twin pairs showed that high intake of dairy products was associated with better short-term memory scores, using the Wechsler memory scale, in men [6]. We previously demonstrated that intake of a dairy product fermented with *Penicillium candidum*, i.e., Camembert cheese, had preventive effects against Alzheimer’s disease pathology in a mouse model [7]. It is concluded that the consumption of dairy products is associated with the prevention of cognitive decline and that some of the ingredients in the dairy products are beneficial to cognitive function [8]; however, the underlying mechanism and responsible agents have not yet been elucidated.

Whey, the supernatant of yogurt and a byproduct of cheese, is rich in protein and consists of β*-*lactalbumin, α-lactoglobulin, immunogloblin, bovine serum albumin, and other minor proteins. Whey protein is a globally consumed food material and is well known for its health benefits, such as its reducing effects on body weight, blood glucose, and blood pressure [9]. In addition, recent clinical trials revealed that whey protein improves cognitive functions and mood status: an intake of 20 g whey protein improved memory performance in stress-vulnerable subjects aged from 18 to 35 years old [10]. Whey protein also reduced depressive symptoms in stress-vulnerable subjects under conditions of stress [11]. In healthy elderly subjects, the intake of 50.5 g whey protein improved delayed paragraph recall [12], but this quantity is not easily consumed in everyday life. We previously demonstrated that some whey peptides, produced through specific enzymatic digestion, improved spatial working memory, episodic memory, and attention in an experiment using scopolamine-induced amnesia model mice and aged mice [13]. The memory improvements were more pronounced in mice which received whey peptide compared with mice which received whey protein: tryptophan-tyrosine (WY)-related peptides, especially the glycine–threonine–tryptophan–tyrosine (GTWY) peptide, were identified for their involvement in the improved cognitive performance. Whey protein did not show any memory improvement effects when used at the same dose as the whey peptide rich in WY-related peptides, suggesting that the whey peptide has greater memory improvement effects than whey protein These studies suggest that intake of whey peptides rich in WY-related peptides could improve cognitive performance in middle aged and older people; however, the effect of whey peptides has not been elucidated. The present study is the first clinical demonstration to evaluate the effects of whey peptides rich in WY-related peptides, which are not whey protein itself but are in fact the result of enzymatic digestion of whey protein, on cognitive function in middle aged and older people in a randomized, placebo-controlled, double-blind, parallel-group comparative study. 

## 2. Materials and Methods 

### 2.1. Subjects 

We recruited 101 healthy Japanese-speaking adults, aged from 45 to 65 years, with a self-awareness of carelessness and forgetfulness; in particular adults that tended to forget the names of people and objects. Subjects with relatively low neuropsychological test scores were preferentially included. Exclusion criteria included: (1) visual or hearing impediments; (2) suspected dementia (Hasegawa Dementia Rating Scale-Revised; HDS-R ≤ 20); (3) anamnesis of cranial nerve disease; (4) current treatment for cognitive function; (5) diagnosis of depressive disorder or depressive symptoms; (6) diagnosed menopausal symptoms or hormone treatment; (7) frequent irregular lifestyle, such as shift work; (8) high habitual consumption of alcohol (>20 g/day); (9) use of cigarettes; (10) experience of the same neuropsychological tests within the prior year; (11) regular consumption of drugs or health foods affecting cognitive functions (>once a week); (12) regular consumption of protein supplements (>once a week); (13) anamnesis of severe disease requiring regular treatment; (14) allergy or sensitivity to milk; (15) pregnancy or breastfeeding; (16) participation in other clinical trials; (17) blood donation within 3 months; (18) unhealthy status as determined during clinical examination; (19) classification of unsuitability by the principal investigator for other reasons. Inclusion and exclusion criteria were checked during the screening session by the questionnaire and clinical examination.

Based on the pilot study, we calculated a sample size of 44 to detect differences of 0.86 in the word recall test with a power of 0.80 and a significance level of 0.05 (two-sided α level). An assumption of a 10% withdrawal rate required at least 50 subjects in each group.

### 2.2. Experimental Supplements

The test formula consisted of 6 tablets containing a total of 1 g of whey peptide, which included 1.6 mg of GTWY peptide; the tablets were ingested by the test group every day for 12 weeks. Whey peptide was purchased from Megmilk Snow Brand Co., Ltd. (Tokyo, Japan). The placebo group ingested tablets substituted with an equivalent amount of maltodextrin every day for 12 weeks. The test and placebo tablets were the same size and shape and were indistinguishable by taste. Using amnesia model mice, we previously evaluated that 1 g of whey peptide, which is much lower than the amount of whey protein in the previous clinical studies (>20 g) [10,11], displayed an equivalent memory improvement to 1.6 mg of GTWY peptide.

### 2.3. Procedures

**Figure 1:**
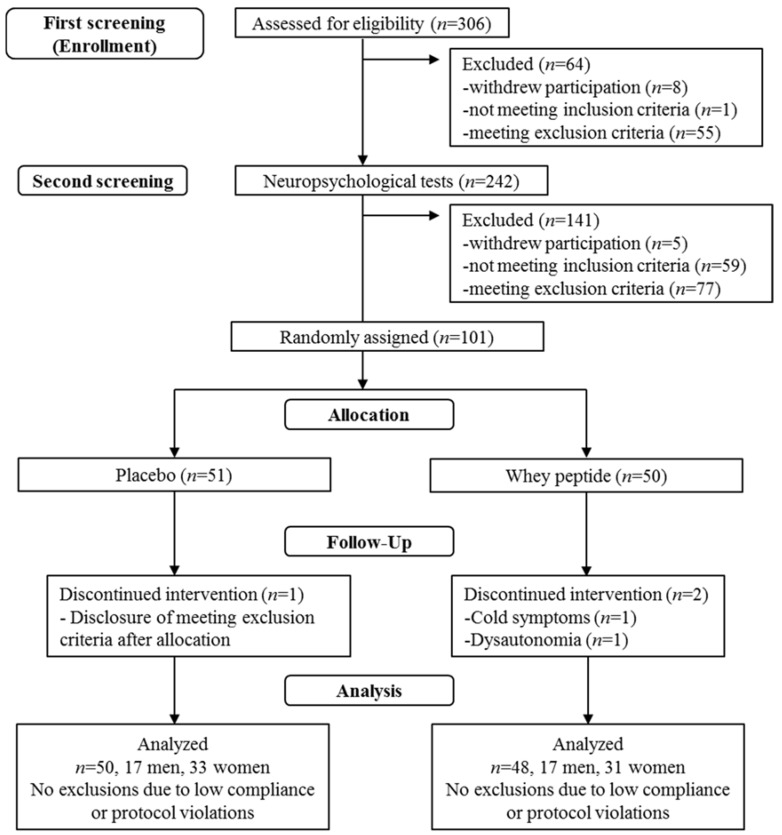
The flow of subjects.

This study was performed using a randomized, placebo-controlled, double-blind, parallel group comparative design. Figure 1 shows the screening procedure for the 101 subjects. Questionnaires for the inclusion/exclusion criteria, the HDS-R, and clinical examinations for safety assessments were performed as part of the first screening step and neuropsychological tests and subjective psychological assessments were performed in the second screening step. Selected subjects were then randomly allocated using the table of random numbers in a 1:1 ratio to the whey peptides group or placebo group under the stratification of the median of the score of the 5 min-delayed word recall and delayed story recall. The person generating the table of random numbers was not involved in determining subject eligibility, data collection, or analysis. Both research staff and subjects were blinded to the group allocation until the completion of data analysis. Neuropsychological tests and subjective psychological assessments were repeated at 6 and 12 weeks of tablet ingestion. Clinical examinations for safety assessment were also performed at 12 weeks. Subjects were instructed to maintain their regular lifestyles and avoid taking any drugs, health functional foods, and protein supplements which could affect the neuropsychological tests during the study. Compliance was monitored by interview, subject diary, and number of ingested tablets. On the day of the neuropsychological tests, subjects were instructed to completely avoid food and beverages containing caffeine and to avoid ingesting other food and beverages, except water, for 4 hours prior to the tests. Subjects were instructed to ingest the study tablets 30 minutes before the start of the neuropsychological tests. The data were collected at Kensyokai Medical Corporation (Osaka, Japan) between June 2017 and December 2017, and the study was conducted by the contract research organization, TTC Co., Ltd. (Tokyo, Japan).

### 2.4. Neuropsychological Tests

Memory function was evaluated using word and story recall tests, and a verbal fluency test (VFT). The word recall test was conducted according to the Hamamatsu Higher Brain Function Scale which assesses short-term memory [14]. Subjects were given seven words and asked to verbally recall them immediately and then 5 and 20 min after they were given. The story recall test was conducted according to the Japanese version of the Rivermead Behavioural Memory Test (RBMT) which also assesses short-term memory [15]. Subjects were given a short story and asked to verbally recall it immediately and 20 min later. The VFT was used to assess long-term memory; subjects were asked to verbally name as many items as they could beginning with “a” (phonemic fluency task) and as many animals as possible (semantic fluency task) in one minute [16]. The Stroop test, digit span, and paced auditory serial addition test (PASAT) were used to evaluate attention and executive functions. For the Stroop test, subjects named words and colors (step 1, read the words printed in black; step 2, read the color of the printed circle; step 3, read words when it has been printed in a different-colored font, e.g., subjects should say red when it has been printed in blue font; step 4, read the print color of words that can be seen in step 3, e.g., subjects should say blue where the word “red” has been printed in blue font). Error numbers and reading time were measured [17]. Digit span (forward) test and PASAT were conducted according to the clinical assessment for attention (CAT) [18]. In the digit span test, which assessed working memory, subjects repeated numbers in increasing spans in forward sequences. The PASAT assessed updating and shifting attention: single digits were introduced every 2 s, and subjects added the new digit to the one immediately prior to it.

### 2.5. Subjective Psychological Assessment

Subjective mental fatigue from the neuropsychological test was assessed using a 100-mm visual analog scale (VAS) before and after the neuropsychological tests. The VAS consisted of a 100-mm line drawn from “not at all” to “most”, and subjects indicated the degree of fatigue at that moment on the line [19]. Previous reports defined high-level fatigue as a greater than 20-mm VAS score [20,21]. The subjective mood status during one week was assessed using the Profile of Mood States (second edition short version; POMS2), which consists of subscales of anger–hostility (AH), confusion–bewilderment (CB), depression–dejection (DD), fatigue–inertia (FI), tension–anxiety (TA), vigor–activity (VA), friendliness (F), and total mood disturbance (TMD). Every POMS2 score was normalized to the T-score which is the score adjusted to the normal distribution (generation average: 50, and standard deviation: 10) and shows the degree of difference from the generation average [22]. To evaluate subjective memory performance, the Japanese version of the everyday memory checklist (EMC), composed of 13 four-point scaled items, was used to assesses memory impairments in an individual’s daily life [23].

### 2.6. Safety Assessment

Safety assessments were performed by clinical examinations and included blood collection (white blood cell (WBC), red blood cell (RBC), hemoglobin (Hb), hematocrit (Ht), platelet (PLT), total protein (TP), albumin (Alb), total bilirubin (TB), direct bilirubin (D-B), indirect bilirubin (I-B), alkaline phosphatase (ALP), aspartate aminotransferase (AST), alanine aminotransferase (ALT), lactate dehydrogenase (LD), γ-glutamyl transpeptidase (γ -GT), total cholesterol (TC), triglyceride (TG), HDL-cholesterol (HDL-C), LDL-cholesterol (LDL-C), urea nitrogen (UN), creatinine (Cr), uric acid (UA), Na, K, CL, glucose (GLU)), urine collection (protein, glucose, uric occult blood), body weight, blood pressure, and pulse measurements. Adverse events were reported in the subject’s diary and supervised by the principal investigator.

### 2.7. Statistical Analysis

The results were expressed as means ± standard deviation (SD). Statistical comparisons were performed using IBM SPSS Statistics 23 (IBM, New York, NY, USA) and Microsoft Excel 2010 (Microsoft, Redmond, WA, USA). Comparisons of all results, except for EMC, were examined with paired *t*-tests (between baseline and after intervention), and unpaired *t*-tests (between groups). EMC results were analyzed using the Wilcoxon signed-rank test (between baseline and after intervention), and Mann–Whitney *u*-tests (between groups). Multiple comparisons were not employed because this study was exploratory.

### 2.8. Ethics and Registration

The study was conducted in accordance with the Declaration of Helsinki, and approved by the ethics committee of Kensyokai Medical Corporation (Osaka, Japan). Written informed consent was obtained from all participants. The study was registered on 5 June 2017 in the database of the University Hospital Medical Information Network (UMIN) prior to enrollment (Registration No. UMIN000027644; Registration title. A study for the effect of intake of ingredients derived from animal on cognitive functions). 

## 3. Results

### 3.1. Baseline Characteristics of the Study Groups

The flow of subjects through the experimental procedure is described in Figure 1. Following the first screening step, 242 subjects were included, and 64 subjects were excluded due to withdrawal of participation (*n* = 8), not meeting inclusion criteria (*n* = 1), suspected dementia (*n* = 3), anamnesis of cranial nerve disease (*n* = 1), high habitual alcohol consumption (*n* = 5), regular consumption of supplements affecting cognitive functions (*n* = 6), anamnesis of severe disease (*n* = 4), classification as unhealthy on clinical examination (*n* = 31), and unsuitability as per the principal investigator (*n* = 5). At the second screening step, 141 subjects were excluded due to withdrawal of participation (*n* = 5), not meeting inclusion criteria (relatively low neuropsychological test score) (*n* = 59), participation in other clinical trials (*n* = 1), anamnesis of severe disease (*n* = 1), and unsuitability as per the principal investigator (*n* = 75). The remaining 101 subjects were randomly allocated into either the whey peptides group or the placebo group for 12 weeks. Two subjects in the whey peptides group withdrew from the study due to whey peptides-unrelated health reasons, and one subject in the placebo group withdrew due to participation in another clinical trial, disclosed after allocation. Finally, 98 subjects completed all tests and were analyzed; their characteristics are shown in Table 1.

Out of 306 subjects who were screened for the inclusion and exclusion criteria, 101 subjects were included in the study. The subjects were randomly allocated into the whey peptide (*n* =50) or placebo (*n* = 51) group. Two subjects in the whey peptide and one subject in the placebo group dropped out of the study during intervention, and thus finally 48 subjects in the whey peptide and 50 subjects in the placebo group were analyzed.

### 3.2. Subjective Psychological Assessment

The baseline scores of the POMS2, VAS, and EMC before intervention are shown in Table 2. There were no significant differences in the POMS2, VAS, and EMC scores between groups before and after intervention, except V-A and F scores. These results suggest that psychological and cognitive fatigue, evaluated by VAS, were induced by the series of neuropsychological tests; however, no psychological improvement between the groups was measured after whey peptide intake.

### 3.3. Neuropsychological Tests (Full Analysis Set (FAS))

The neuropsychological test measurements at weeks 0 (baseline), 6, and 12, in addition to the changes from baseline are shown in Table 3 (memory) and Table 4 (attention and executive functions). The changes in VFT (words beginning with “a”) at 12 weeks of intervention from baseline tended to be higher in the whey peptide group compared with the placebo group (*p* = 0.094). In the VFT (animal), the scores were significantly increased at 6 weeks of intervention compared to baseline in the whey peptide group (*p* = 0.012) but there were no significant changes in the placebo group. These results suggest that whey peptide intake may affect long term memory retrieval rather than short term memory, however, there were no significant differences between the groups in the neuropsychological tests.

### 3.4. Subgroup Analysis by the Degree of Fatigue

The change in VAS scores between before and after the neuropsychological tests indicated that the series of cognitive tasks induced psychological fatigue in subjects. The VAS score levels differed among the subjects, and therefore subjects were classified into two subgroups of high-level and low-level fatigue, similar to previous reports [20,21]. Previous reports defined high-level fatigue as a VAS greater than 20 mm.

**Figure 2:**
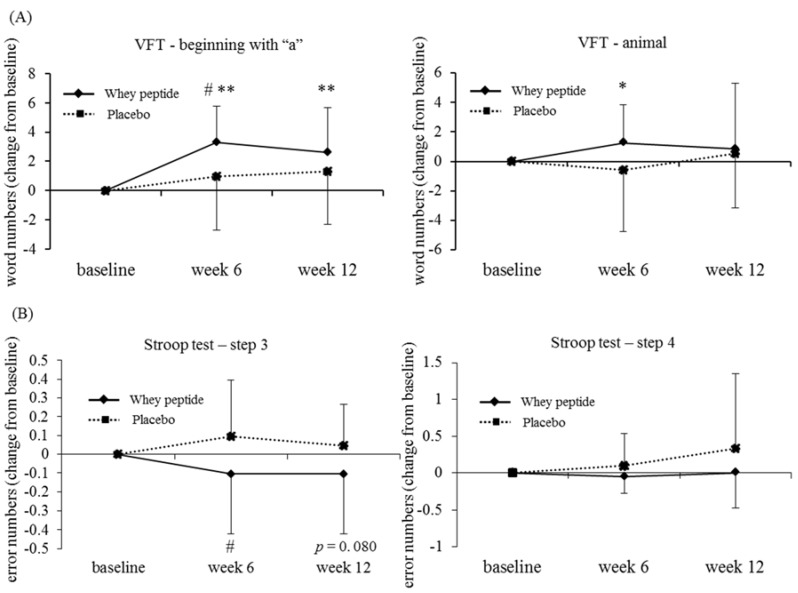
Changes of word numbers in the VFT (**A**) and error numbers in the Stroop test—step 3 (**B**) from baseline in the high fatigue subgroup, classified by the VAS. The solid line shows the whey peptide group (*n* = 19) and the dotted line shows the placebo group (*n* = 21). The data represent mean, and error bars indicate SD. The *p*-values and # show between group differences, performed using unpaired *t*-tests, # *p* < 0.05. * *p* < 0.05; ** *p* < 0.01 performed using paired *t*-tests (vs. baseline).

In the high-level fatigue subgroup, the increase in the number of recalled words beginning with “a” in the VFT at 6 weeks from baseline was significantly higher in the whey peptide group compared with the placebo group (*p* = 0.023). In addition, the number of recalled words beginning with “a” at 6 and 12 weeks and the number of animal words recalled at 6 weeks were significantly increased from baseline in the whey peptide group, whereas the scores did not change in the placebo group (Table 5 and Figure 2A).

The reduction in error numbers in the Stroop test (step 3), was significantly lower at 6 weeks in the high fatigue whey peptide group compared with the high fatigue placebo group (*p* = 0.047); at 12 weeks the reduction in error numbers tended to be lower in the whey peptide group compared with the placebo group (*p* = 0.080, Table 5 and Figure 2B). These results suggest that whey peptide intake for 6 weeks improves long-term memory retrieval, attention, and executive functions in subjects vulnerable to psychological fatigue.

**Figure 3:**
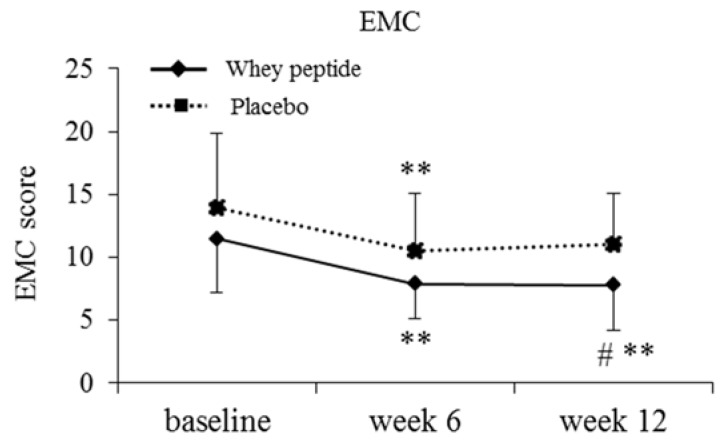
Changes in the EMC scores in the high fatigue subgroup, classified using the VAS. The solid line shows the whey peptide group (*n* = 19) and the dotted line shows the placebo group (*n* = 21). The data represent mean, and error bars indicate SD. # *p* < 0.05 between groups performed using the Mann–Whitney *u*-test. ** *p* < 0.01 performed using the Wilcoxon signed-rank test (vs. baseline).

In addition to the neuropsychological tests, the EMC scores, which give a subjective measure of memory failure in everyday life, were significantly lower in the high fatigue whey peptide group compared with the high fatigue placebo group at 12 weeks. In addition, the EMC score was significantly decreased compared to baseline in the whey peptide group at 12 weeks, but not in the placebo group (Figure 3).

In the low-level fatigue subgroup, there were no significant differences between the whey peptide and placebo groups in the neuropsychological test results, except the word recall test: immediate recall was significantly higher in the whey peptide group compared with the placebo group at 6 weeks (5.2 (whey peptide group), 4.8 (placebo), *p* = 0.040, data not shown).

**Figure 4:**
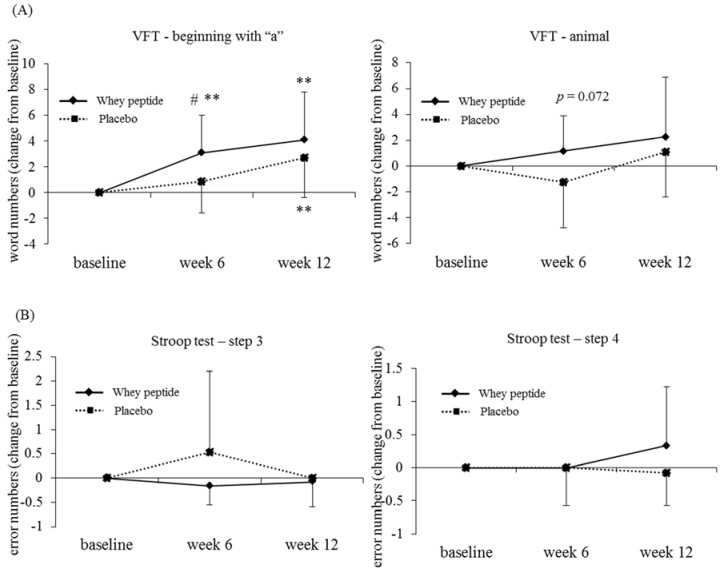
Changes of word numbers in the VFT and error numbers in the Stroop test from baseline in the high-fatigue subgroup, classified using POMS2. The solid line shows the whey peptide group (*n* = 12) and the dotted line shows the placebo group (*n* = 13). The data represent mean, and error bars indicate SD. The *p*-values and # show between group differences, performed using unpaired *t*-tests, # *p* < 0.05. ** *p* < 0.01 performed using paired *t*-tests (vs. baseline).

Subjects were also classified into fatigue-level subgroups using the FI scores from the POMS2 test, which indicates fatigue level in subjects in the week that tests are performed. The average test score is set as 50, therefore subjects with an FI score more than or equal to 50 at baseline were defined as the high fatigue subgroup. The changes in number of recalled words beginning with “a” and animal names were significantly higher or tended to be higher in the high fatigue whey peptide group compared with the high fatigue placebo group at 6 weeks (*p* = 0.047 and *p* = 0.072, respectively) (Table 6 and Figure 4).

### 3.5. Safety Assessment and Compliance

In order to evaluate the safety of whey peptide used in this study, we conducted safety assessments using subject diaries in 101 subjects (whole subjects after allocation) and clinical assessments in 98 subjects (final number of subjects completing the analysis): 74 adverse events occurred in the placebo group (30 subjects/51 subjects), and 63 adverse events occurred in the whey peptide group (25 subjects/50 subjects). None of the adverse events were severe and were judged to have no association with whey peptide by the principal investigator. Slight changes to some of the clinical assessment values from baseline were detected but these were judged to have no clinical significance by the principal investigator. Compliance with this trial was high, with a tablet consumption rate of 91.7–102.4%. The changes in clinical assessments are shown in supplementary Tables S1–S3.

## 4. Discussion

This is the first reported evaluation of the effects of whey peptide on cognitive performance in middle- and older-aged volunteers in a randomized, double-blind, placebo-controlled trial. The low dropout rate (3%) during the interventions and no exclusions during the analysis increases the reliability of this study. Whey peptide intake tended to improve the VFT score, reflecting fluent semantic memory retrieval in middle- and older-aged healthy subjects with self-awareness of cognitive decline. In the full analysis set, there was no significant improvement in neuropsychological tests between the groups.

Subgroup analysis, stratified for subjective fatigue, revealed that the intakes of whey peptides for 6 weeks improved the fluent semantic memory retrieval (VFT), and attention and executive function (Stroop test) in the subjects with high fatigue. In these tests, the improvements were significant after 6 weeks of whey peptide intake but not significant after 12 weeks. It is suggested that repeated memory and attention assessments might increase the learning effects at week 12, resulting in a reduction in the difference between placebo and whey peptide group at week 12 compared with week 6. According to previous reports [20,21], subjects with high fatigue levels were classified as those with over 20-mm changes in their VAS before and after neuropsychological testing at week 0 of the intervention. Since subjects are required to concentrate for about one hour during the neuropsychological tests, those experiencing fatigue are considered to be vulnerable to psychological or cognitive fatigues. In addition to the neuropsychological test results, subjective memory conditions in daily life, measured by EMC, were also improved in the high fatigue subgroup. The fatigue levels indicated by the VAS score in the present study reflect the fatigue induced by the neuropsychological test, which indicates a short-term acute condition; the fatigue levels indicated by POMS2 reflect the fatigue in the subject’s daily life for as recently as one week, which indicates a long-term condition. Whey peptide intake could improve fluent semantic memory retrieval, attention and executive function in subjects with both acute and chronic psychological fatigue. However, it should be noted that VAS and POMS2 are subjective scales. Subjective fatigue is affected by multiple physical and psychological conditions, so, we could not clearly assert “fatigue”, and further study will elucidate this issue.

In the present study, whey peptide intake improved the phonemic verbal fluency of the VFT and error numbers in the Stroop test. VFT is often included in clinical practice to diagnose the cognitive impairment of neurodegenerative disorders, such as Alzheimer’s disease, and in research to measure verbal ability and lexical retrieval ability [24]. Phonemic verbal fluency, including words beginning with a specific letter, is closely associated with the frontal cortex, especially the dorsolateral prefrontal cortex (dlPFC); sematic words, including objects such as animals, are associated with the temporal cortex [25,26]. Neuroimaging studies revealed that phonemic fluency tests activate the neurons in the dlPFC [25]. The Stroop test is used to evaluate inhibition of executive function, which is composed of inhibition, updating/shifting and working memory [27]. In the present study, the functions of inhibition, updating/shifting, and working memory were measured using the Stroop test, PASAT, and digit span, respectively. Inhibition of executive function is closely associated with conditions of the prefrontal cortex, especially the dlPFC, and anterior cingulate cortex (ACC). Functional magnetic resonance imaging revealed that dlPFC and ACC play important roles in the implementation of control and performance monitoring in incongruent stimuli, respectively [28]. The evidence suggests that whey peptide intake may improve the function of the frontal cortex, especially the dlPFC, and ACC. Psychological and cognitive fatigue induced by cognitive tasks in the present study are common symptoms in neurological disorders (e.g., dementia, mild traumatic brain injury) and aging, which are associated with executive functions and conditions of the PFC and ACC [29,30,31]. Subjects with high fatigue levels might be sensitive to the effects of whey peptides in certain neuropsychological tests, e.g., the VFT and Stroop test which are related to PFC and ACC performance.

We previously demonstrated that the whey peptide used in the present study improves long-term episodic memory and spatial working memory in amnesia model mice and aged mice and identified GTWY peptides as an ingredient to improve cognitive function [13]. Short-term oral administration of GTWY peptides increased dopamine levels in the brains of mice. The dopaminergic system in the PFC is crucial for cognitive function, including attention, executive function, learning, and memory function [32,33]; intake of whey peptide rich in GTWY peptides might improve the fluency and executive functions via dopaminergic activation in the PFC. In addition to dopaminergic activation, some whey peptides have anti-oxidant and anti-inflammatory activity [34,35]. Thus, reduction of oxidative stress and inflammation might contribute to the beneficial effects of whey peptide in this study. Further clinical studies to evaluate the effects of whey peptides on cerebral activity, oxidative stress, or inflammatory markers will be conducted to elucidate the underlying mechanisms.

There are some limitations in this study: firstly, the fatigue scales evaluated in the present study are subjective methods, which might be affected by other psychological conditions other than fatigue; further studies measuring biochemical fatigue markers (e.g., salivary amylase) should be conducted in order to distinguish fatigue from other psychological conditions. Secondly, multiple comparisons were not employed, so there is a risk of α-errors. Further studies should confirm the results obtained in this study. Thirdly, we could not dissociate the chronic effects of whey peptide from the acute effects: further studies are required to dissociate these effects. 

## 5. Conclusions

In the present study, the effects of daily whey peptide intake on cognitive functions in healthy middle- and older-aged people were evaluated. It is suggested that whey peptide improves some cognitive functions in people with a high level of subjective fatigue. Our results support previous epidemiological and preclinical findings which suggested that intake of whey peptide in daily life might be beneficial to cognitive function.

## Figures and Tables

**Table 1:** Characteristics of the analyzed subjects at baseline (week 0).

Characteristics	Placebo (*n* = 50)	Whey Peptide (*n* = 48)	*p*-Value
Age	51.8 ± 5.2	52.3 ± 4.3	0.568
Male/female	17/33	17/31	1.000
Body mass index (kg/m^2^)	21.35 ± 2.04	21.11 ± 2.10	0.564
Education years	14.1 ± 1.8	14.5 ± 2.0	0.406
HDS-R score	28.4 ± 1.5	28.5 ± 1.8	0.678

Data are the mean ± SD. The *p*-values were calculated using unpaired *t*-tests, except for the male/female *p*-value which was calculated using the χ^2^ test. HDS-R: Hasegawa Dementia Rating Scale-Revised.

**Table 2:** Subjective psychological assessments at baseline (week 0).

Psychological Status	Placebo (*n* = 50)	Whey Peptide (*n* = 48)	*p*-Value
**POMS2**			
AH	45.9 ± 9.1	45.5 ± 8.7	0.842
CB	48.8 ± 7.6	48.2 ± 8.4	0.707
DD	46.9 ± 6.0	46.3 ± 7.3	0.663
FI	45.2 ± 7.9	44.2 ± 8.9	0.547
TA	47.8 ± 7.4	46.4 ± 7.9	0.360
VA	47.2 ± 8.3	53.0 ± 10.2	0.002
F	48.8 ± 8.9	53.3 ± 9.5	0.019
TMD	47.2 ± 6.9	45.0 ± 8.6	0.159
VAS (after-before)	19.1 ± 16.1	20.6 ± 18.1	0.661
EMC	13.0 ± 4.8	12.8 ± 4.6	0.826

Data are presented as the mean ± SD. Unpaired *t*-tests were performed for the POMS2 and VAS. The Mann–Whitney *u*-test was performed for EMC. POMS2: Profile of Mood States (second edition short version); VAS: visual analog scale; EMC: everyday memory checklist; AH: anger–hostility; CB: confusion–bewilderment; DD: depression–dejection; FI: fatigue–inertia; TA: tension–anxiety; VA: vigor–activity; F: friendliness; TMD: total mood disturbance.

**Table 3:** Changes in neuropsychological tests assessing memory function.

Test		Group	Week 0 (Baseline)	Week 6	Week 12
**Word recall**					
Immediate recall	Score	P	4.2 ± 0.9	4.8 ± 0.9 **	5.1 ± 1.1 **
		W	4.4 ± 1.0	5.1 ± 0.9 **	5.2 ± 1.0 **
	Changes from baseline	P		0.7 ± 1.3	0.9 ± 1.1
		W		0.8 ± 1.2	0.8 ± 1.3
5-min delayed recall	Score	P	3.6 ± 1.1	4.9 ± 1.3 **	5.3 ± 1.2 **
		W	3.7 ± 1.1	4.7 ± 1.5 **	5.1 ± 1.4 **
	Changes from baseline	P		1.3 ± 1.4	1.7 ± 1.3
		W		1.0 ± 1.9	1.4 ± 1.7
20-min delayed recall	Score	P	4.3 ± 1.2	5.3 ± 1.3 **	5.6 ± 1.4 **
		W	4.2 ± 1.2	5.1 ± 1.6 **	5.4 ± 1.4 **
	Changes from baseline	P		1.0 ± 1.8	1.3 ± 1.6
		W		0.9 ± 1.8	1.2 ± 1.7
**Story recall**					
Immediate recall	Score	P	15.93 ± 2.53	17.65 ± 3.03 **	17.56 ± 2.77 **
		W	15.51 ± 2.55	16.58 ± 2.64 *	16.92 ± 2.52 **
	Changes from baseline	P		1.72 ± 3.71	1.63 ± 3.63
		W		1.07 ± 3.08	1.41 ± 3.38
20-min delayed recall	Score	P	14.39 ± 3.05	16.59 ± 3.45 **	16.63 ± 2.81 **
		W	14.44 ± 2.50	15.88 ± 3.00 **	16.83 ± 2.53 **
	Changes from baseline	P		2.20 ± 3.83	2.24 ± 3.65
		W		1.44 ± 3.51	2.40 ± 3.66
**VFT (verbal fluency test)**					
Beginning with “a”	Score	P	12.1 ± 3.3	13.1 ± 3.6 *	13.7 ± 3.8 **
		W	11.4 ± 4.1	13.2 ± 3.9 **	14.1 ± 4.1 **
	Changes from baseline	P		1.0 ± 2.8	1.7 ± 3.1
		W		1.9 ± 3.3	2.8 ± 3.1
Animal	Score	P	19.5 ± 4.4	19.6 ± 4.7	20.9 ± 3.9 *
		W	18.3 ± 4.1	19.4 ± 4.0 *	20.1 ± 4.6 **
	Changes from baseline	P		0.2 ± 3.7	1.4 ± 3.9
		W		1.2 ± 3.2	1.9 ± 3.9

Data are presented as the mean ± SD for the placebo (P) (*n* = 50) and whey peptide (W) groups (*n* = 48). * *p* < 0.05, ** *p* < 0.01, performed by paired *t*-tests (vs. baseline).

**Table 4:** Changes in neuropsychological tests assessing attention and executive functions.

Test		Group	Week 0 (Baseline)	Week 6	Week 12
**Stroop test**					
Step 3 error numbers	Score	P	0.0 ± 0.1	0.2 ± 0.9	0.1 ± 0.3
		W	0.1 ± 0.3	0.0 ± 0.2	0.1 ± 0.3
	Changes from baseline	P		0.1 ± 0.9	0.1 ± 0.3
		W		0.0 ± 0.4	0.0 ± 0.5
Step 3 reading time (second)	Score	P	45.6 ± 7.1	42.9 ± 8.1 **	41.0 ± 7.5 **
		W	44.8 ± 10.7	42.4 ± 9.6 **	40.9 ± 7.8 **
	Changes from baseline	P		−2.7 ± 6.0	−4.6 ± 6.9
		W		−2.4 ± 5.8	−3.9 ± 8.2
Step 4 error numbers	Score	P	0.1 ± 0.3	0.1 ± 0.4	0.2 ± 0.7
		W	0.2 ± 0.5	0.2 ± 0.4	0.2 ± 0.6
	Changes from baseline	P		0.0 ± 0.4	0.1 ± 0.7
		W		0.0 ± 0.6	0.0 ± 0.6
Step 4 reading time (second)	Score	P	59.5 ± 15.4	53.5 ± 9.9 **	50.8 ± 12.0 **
		W	57.2 ± 11.4	52.0 ± 10.1 **	49.7 ± 8.5 **
	Changes from baseline	P		−6.1 ± 10.8	−8.7 ± 12.4
		W		−5.2 ± 6.8	−7.5 ± 7.1
**Digit Span**					
Spans	Score	P	6.1 ± 1.1	6.4 ± 1.2 *	6.6 ± 1.4 **
		W	6.2 ± 1.2	6.3 ± 1.1	6.6 ± 1.1*
	Changes from baseline	P		0.3 ± 1.0	0.5 ± 1.1
		W		0.1 ± 1.1	0.4 ± 1.1
**PASAT (paced auditory serial addition test)**					
Accuracy (%)	Score	P	68.8 ± 16.0	77.4 ± 16.6 **	83.1 ± 11.7 **
		W	70.0 ± 16.0	76.4 ± 14.8 **	78.4 ± 19.6 **
	Changes from baseline	P		8.6 ± 12.3	14.2 ± 11.8
		W		6.4 ± 12.7	8.4 ± 18.1

Data are presented as the mean ± SD for the placebo (P) (*n* = 50) and whey peptide (W) (*n* = 48) groups. * *p* < 0.05, ** *p* < 0.01, performed by paired *t*-tests (vs. baseline).

**Table 5:** Changes of word numbers in VFT and error numbers in the Stroop test from baseline (week 0) in the high and low fatigue subgroups, classified using the VAS.

Test		Group	Week 6	Week 12	Week 6	Week 12
**VFT**			**High fatigue**		**Low fatigue**	
Beginning with “a”	Changes from baseline	P	1.0 ± 3.7	1.3 ± 3.6	1.1 ± 2.2 *	2.0 ± 2.8 **
		W	3.3 ± 2.5 **^,#^	2.6 ± 3.0 **	0.9 ± 3.5	2.8 ± 3.2 **
Animal	Changes from baseline	P	−0.6 ± 4.2	0.5 ± 3.7	0.7 ± 3.3	2.0 ± 3.9 **
		W	1.3 ± 2.6 *	0.8 ± 4.5	1.1 ± 3.6	2.5 ± 3.4 **
**Stroop test**			**High fatigue**		**Low fatigue**	
Step 3 error numbers	Changes from baseline	P	0.1 ± 0.3	0.0 ± 0.2	0.2 ± 1.1	0.1 ± 0.4
		W	−0.1 ± 0.3 ^#^	−0.1 ± 0.3	0.0 ± 0.4	0.1 ± 0.5
Step 4 error numbers	Changes from baseline	P	0.1 ± 0.4	0.3 ± 1.0	0.0 ± 0.4	0.0 ± 0.4
		W	−0.1 ± 0.2	0.0 ± 0.5	0.0 ± 0.7	0.0 ± 0.7

Data are presented as the mean ± SD for the placebo (P) (*n* = 21) and whey peptide (W) (*n* = 19) groups classified into the high fatigue subgroup, and placebo (*n* = 29) and whey peptide (*n* = 29) groups in the low fatigue subgroup. ^#^ *p* < 0.05 performed using unpaired *t*-tests. * *p* < 0.05, ** *p* < 0.01, performed using paired *t*-tests (vs. baseline).

**Table 6:** Changes of word numbers in the VFT and error numbers in the Stroop test from baseline (week 0) in the high and low fatigue subgroups, classified using the POMS2 FI score.

Test		Group	Week 6	Week 12	Week 6	Week 12
**VFT**			**High fatigue**		**Low fatigue**	
Beginning with “a”	Changes from baseline	P	0.8 ± 2.4	2.7 ± 3.1 **	1.1 ± 3.0 *	1.3 ± 3.1 *
		W	3.1 ± 2.9 **^,#^	4.1 ± 3.7 **	1.4 ± 3.4 *	2.3 ± 2.8 **
Animal	Changes from baseline	P	−1.2 ± 3.5	1.1 ± 3.5	0.7 ± 3.7	1.5 ± 4.0 *
		W	1.2 ± 2.7	2.3 ± 4.6	1.2 ± 3.3 *	1.7 ± 3.7 **
**Stroop test**			**High fatigue**		**Low fatigue**	
Step 3 error numbers	Changes from baseline	P	0.5 ± 1.7	0.0 ± 0.0	0.0 ± 0.2	0.1 ± 0.4
		W	−0.2 ± 0.4	−0.1 ± 0.5	0.0 ± 0.3	0.0 ± 0.4
Step 4 error numbers	Changes from baseline	P	0.0 ± 0.6	−0.1 ± 0.5	0.0 ± 0.4	0.2 ± 0.8
		W	0.0 ± 0.0	0.3 ± 0.9	−0.1 ± 0.7	−0.1 ± 0.5 ^#^

Data are presented as the mean ± SD for the placebo (P) (*n* = 13) and the whey peptide (W) (*n* = 12) groups classified into the high fatigue subgroup, and placebo (*n* = 37) and the whey peptide (*n* = 36) groups in the low fatigue subgroup. ^#^ *p* < 0.05 performed using the unpaired *t*-test. * *p* < 0.05, ** *p* < 0.01 performed using the paired *t*-test (vs. baseline).

## Supplementary Materials

The following are available online at http://www.mdpi.com/2072-6643/10/7/899/s1, Table S1: Clinical assessment of the changes in blood parameters, Table S2: Clinical assessment of the changes in urine parameters, Table S3: Changes in weight, blood pressure, and pulse.
