# Supplementary material for: Effect of Supplementation of a Whey Peptide Rich in Tryptophan-Tyrosine-Related Peptides on Cognitive Performance in Healthy Adults: A Randomized, Double-Blind, Placebo-Controlled Study
Source: Nutrients. 2018 Jul 13;10(7):899. doi: 10.3390/nu10070899 (PMC6073406; doi:10.3390/nu10070899)
Supplement: Supplementary file 1 [file nutrients-10-00899-s001.pdf]

Supplementary table 1. Clinical assessment of the changes in blood parameters

Evaluation items	Unit	Group	Baseline	Week 12
WBC	/μL	P	5,838.0 ± 1,400.6	6,130.0 ± 1,384.1
		W	5,504.2 ± 1,528.4	6,227.1 ± 1,838.0**
RBC	×10^4 /μL	P	446.1 ± 34.9	457.0 ± 39.3**
		W	450.9 ± 33.8	463.7 ± 34.6**
Hb	g/dL	P	13.46 ± 1.12	13.93 ± 1.25**
		W	13.80 ± 0.97	14.18 ± 0.98**
Ht	%	P	42.36 ± 3.00	43.27 ± 3.36**
		W	43.23 ± 2.94	44.05 ± 2.83**
PLT	×10^4 /μL	P	26.90 ± 5.62	28.66 ± 5.84**
		W	26.88 ± 5.63	29.04 ± 6.01**
TP	g/dL	P	7.26 ± 0.41	7.37 ± 0.37*
		W	7.22 ± 0.39	7.38 ± 0.38**
Alb	g/dL	P	4.38 ± 0.21	4.48 ± 0.24**
		W	4.40 ± 0.20	4.50 ± 0.25**
UN	mg/dL	P	14.10 ± 3.51	13.04 ± 3.43*
		W	13.61 ± 3.85	13.39 ± 3.09
Cr	mg/dL	P	0.73 ± 0.17	0.71 ± 0.16*
		W	0.74 ± 0.16	0.72 ± 0.16
UA	mg/dL	P	4.93 ± 1.28	4.70 ± 1.27**
		W	4.87 ± 1.21	4.74 ± 1.12
TC	mg/dL	P	215.6 ± 26.8	223.7 ± 32.4**
		W	217.0 ± 34.8	224.8 ± 32.3**
LDL-C	mg/dL	P	122.4 ± 25.5	128.3 ± 29.9**
		W	124.5 ± 28.9	130.3 ± 26.2*
HDL-C	mg/dL	P	70.5 ± 15.9	72.8 ± 15.5*
		W	71.1 ± 17.6	73.9 ± 18.6**
TG	mg/dL	P	101.7 ± 47.6	113.7 ± 78.2
		W	84.7 ± 47.6	88.5 ± 53.2
TB	mg/dL	P	0.67 ± 0.20	0.71 ± 0.20
		W	0.75 ± 0.22	0.77 ± 0.25
D-B	mg/dL	P	0.11 ± 0.04	0.09 ± 0.05*
		W	0.13 ± 0.05	0.10 ± 0.05**
I-B	mg/dL	P	0.56 ± 0.17	0.61 ± 0.19
		W	0.61 ± 0.18	0.67 ± 0.23*
ALP	U/L	P	204.3 ± 58.5	218.3 ± 65.0**
		W	203.4 ± 48.4	217.5 ± 53.8**
AST（GOT）	U/L	P	19.5 ± 4.3	21.9 ± 7.2**
		W	20.2 ± 4.6	20.8 ± 5.9
ALT（GPT）	U/L	P	15.7 ± 5.4	18.5 ± 10.4*
		W	16.7 ± 8.1	17.6 ± 9.3
γ-GT（γ-GTP）	U/L	P	22.5 ± 14.9	26.9 ± 28.7
		W	21.6 ± 11.4	23.3 ± 13.0*
LD	U/L	P	175.1 ± 36.6	182.4 ± 36.4**
		W	173.6 ± 23.5	176.2 ± 24.7
Na	mEq/L	P	141.8 ± 1.8	141.4 ± 1.6
		W	142.3 ± 1.7	142.0 ± 1.8
K	mEq/L	P	4.32 ± 0.29	4.34 ± 0.36
		W	4.34 ± 0.30	4.35 ± 0.28
CL	mEq/L	P	105.4 ± 2.1	104.6 ± 2.3**
		W	105.6 ± 2.0	104.6 ± 2.2**
GLU	mg/dL	P	80.3 ± 7.1	81.4 ± 6.3
		W	80.7 ± 6.7	80.9 ± 11.7

Data are presented as the mean ± SD for the placebo (P) (n = 50) and whey peptide (W) groups (n =48).
* $p < 0.05$, ** $p < 0.01$, performed by paired *t*-tests (v.s. baseline).

Supplementary table 2. Clinical assessment of the changes in urine parameters

Evaluation items	Evaluation	Baseline		Week 12	
		P	W	P	W
Urine protein (Qualitative evaluation)	(-)	47	46	46	42
	(+-)	1	1	3	5
	(+)	2	1	0	1
	(2+)	0	0	1	0
	(3+)	0	0	0	0
Urine glucose (Qualitative evaluation)	(-)	50	48	50	47
	(+-)	0	0	0	0
	(+)	0	0	0	0
	(2+)	0	0	0	0
	(3+)	0	0	0	1
Uric occult blood (Qualitative evaluation)	(-)	42	36	40	39
	(+-)	4	8	5	5
	(+)	2	1	2	2
	(2+)	2	1	2	0
	(3+)	0	2	1	2

Number of people are presented for the placebo (P) (n = 50) and whey peptide (W) groups (n =48).

Supplementary table 3. Changes in weight, blood pressure, and pulse

Evaluation items	Unit	Group	Baseline	Week 6	Week 12
Weight	kg	P	56.56 ± 9.99	56.97 ± 10.14*	57.22 ± 10.08**
		W	55.50 ± 8.01	55.90 ± 8.14*	56.04 ± 8.15**
BMI	kg/m^2	P	21.35 ± 2.04	21.51 ± 2.15*	21.61 ± 2.17**
		W	21.11 ± 2.10	21.26 ± 2.13*	21.31 ± 2.11**
Systolic pressure	mmHg	P	114.7 ± 14.3	112.4 ± 12.2	113.5 ± 14.2
		W	116.4 ± 15.1	112.3 ± 14.7**	112.0 ± 12.2**
Diastolic pressure	mmHg	P	69.8 ± 10.7	70.1 ± 9.8	70.5 ± 10.4
		W	69.7 ± 9.7	71.2 ± 10.6	70.3 ± 9.5
Pulse	bpm	P	66.5 ± 9.2	69.8 ± 9.0**	70.3 ± 8.6**
		W	67.6 ± 8.7	71.2 ± 9.0**	72.9 ± 11.0**

Data are presented as the mean ± SD for the placebo (P) (n = 50) and whey peptide (W) groups (n =48).
* $p < 0.05$, ** $p < 0.01$, performed by paired *t*-tests (v.s. baseline).
